# From Forest Extraction to Digital Markets: Illegal Macaw Trade and Adaptive Monitoring for Forest Wildlife Resilience

**DOI:** 10.3390/ani16152430

**Published:** 2026-08-06

**Authors:** Christopher Morales

**Affiliations:** Department of Pure and Applied Sciences, Florida SouthWestern State College, Fort Myers, FL 33919, USA; christopher.morales@fsw.edu

**Keywords:** macaw, illegal wildlife trade, parrot trade, CITES, captive breeding, laundering risk, online wildlife trade, forest resilience, adaptive monitoring, conservation enforcement

## Abstract

Illegal wildlife trade contributes to biodiversity loss, but trade in forest-dependent parrots is often examined through disconnected evidence streams, including field observations, declared trade records, seizures, online advertisements, and animal-welfare cases. Macaws provide a useful focal group because many species combine high market visibility with delayed maturity, long life spans, low reproductive output, and dependence on suitable nesting cavities. This structured narrative review synthesizes 80 sources, including peer-reviewed studies, CITES resources, official reports, policy documents, and the conservation literature. Sources were coded by taxonomic focus, geographic region, trade-chain level, source type, risk domain, and monitoring method and classified as direct macaw evidence, transferable parrot evidence, or broader wildlife-trade evidence. Direct studies, including long-term research from Peru, show that extraction may interact with habitat loss, nest-site limitation, chick survival, recruitment constraints, and population structure, although species-specific estimates of illegal extraction remain limited. Peruvian market studies further demonstrate the importance of domestic trade outside international reporting systems. CITES and seizure records can identify declared trade patterns and enforcement contacts but cannot provide complete measures of illegal activity. Captive-bred claims require documentary, biological, and, where appropriate, genetic verification, while digital markets disperse trade signals across public listings, seller networks, temporary posts, and private communication. The review proposes a multiscale adaptive monitoring framework connecting ecological evidence, trade and enforcement records, digital-market observations, source verification, and management response. Available evidence supports treating illegal and poorly regulated macaw trade as a plausible conservation stressor, but stronger species-specific datasets are needed to estimate prevalence, demographic effects, and intervention outcomes.

## Simple Summary

Macaws are highly visible forest parrots that are frequently traded as exotic pets. Removing eggs, chicks, or adults from wild populations can create welfare and conservation concerns because many macaws are long-lived, reproduce slowly, form lasting social bonds, and depend on suitable nesting cavities. Some macaws also disperse large seeds, so population declines may affect forest regeneration. This review examines illegal and poorly regulated macaw trade as a problem extending from forest extraction to physical and online markets. It distinguishes direct research on macaws from evidence concerning other parrots and the broader wildlife trade. It also explains the limitations of official trade records, seizures, online advertisements, and captive-bred claims when these sources are interpreted separately. The review proposes an adaptive monitoring framework that connects field ecology, trade records, digital-market observations, source verification, and conservation responses. The framework is intended to guide further research and coordinated monitoring, not to establish that any particular bird, seller, breeder, advertisement, or trade pathway is illegal.

## 1. Introduction

Illegal wildlife trade can reduce wild populations, alter demographic structure, weaken recruitment, and disrupt ecological relationships, particularly when extraction affects long-lived species with low reproductive rates [1,2,3,4,5,6,7]. The World Wildlife Crime Report 2024 concluded that wildlife trafficking persists despite sustained enforcement efforts and that seizures recorded from 2015 to 2021 involved thousands of plant and animal species across numerous countries and territories [8]. Seizure records document enforcement contact rather than the full prevalence of illegal activity, while CITES records primarily describe declared international trade [8,9,10,11,12]. Field observations, market surveys, trade records, seizures, and online advertisements therefore provide distinct but incomplete perspectives on wildlife extraction and commerce [8,9,10,11,12,13,14,15,16,17,18,19,20]. Regional and taxonomically diverse case studies further show that wildlife trade can span tourism settings, traditional markets, digital platforms, and transnational routes [21,22,23,24,25].

Macaws provide a useful focal group because their longevity, delayed reproduction, social behavior, and dependence on suitable nesting resources may limit the ability of wild populations to compensate for removals [5,6,7,26,27,28,29,30]. Research across traded birds also indicates that coloration may influence market targeting, providing additional context for macaw desirability [31]. Long-term research in southeastern Peru has examined Scarlet Macaw nest-site selection, artificial-nest use, reproductive performance, brood reduction, chick mortality, foster parenting, and supplemental feeding [32,33,34]. Studies of Hyacinth Macaws and Great Green Macaws have likewise identified habitat availability, protected-area coverage, nest-site characteristics, reproductive success, and population genetic structure as important conservation variables [26,27,28,35]. These studies indicate that egg or chick removal reduces immediate reproductive output, whereas adult removal may eliminate future reproductive contributions in populations whose growth depends strongly on adult survival [5,6,7,26,27,28,32,33,34]. Macaws and other parrots may also contribute to seed dispersal and plant regeneration, extending the potential ecological consequences of population decline beyond the loss of individual birds [36]. These population and ecosystem concerns also align with broader international priorities for sustainable wildlife use and biodiversity recovery [37,38].

Research from Peru also demonstrates that domestic markets must be considered alongside international trade. Surveys conducted across markets in multiple Peruvian cities documented thousands of parrots in trade, including threatened species and macaws [39]. More recent monitoring at the Belén and Modelo markets in Iquitos established a contemporary baseline for wildlife availability, species composition, and trade practices [17]. Together, these studies show that wildlife commerce can occur substantially within source countries and outside international reporting systems [17,39]. Their findings remain geographically and temporally bounded, however, and cannot establish current trade prevalence across all Peruvian regions, macaw taxa, or supply routes [17,39].

Foundational and long-term research provides additional context for interpreting these risks. In southeastern Peru, comparative research has documented species-specific differences in cavity use and reproductive success among Scarlet, Red-and-green, and Blue-and-yellow Macaws, complementing subsequent studies of artificial nests, brood reduction, and chick-management interventions [33,34,40]. Long-term monitoring in Costa Rica has evaluated population and recruitment responses to nest protection, anti-poaching, artificial nests, environmental education, and stakeholder coordination [41], while reintroduction projects in Peru and Costa Rica have documented post-release survival and factors associated with successful establishment [42]. Research on Lear’s Macaws in Brazil further demonstrates that total population counts may obscure the smaller fraction of individuals actively breeding and variation in reproductive performance [43]. At the institutional level, Peruvian research shows that responses to domestic wildlife-pet trade can involve outreach, advocacy, livelihood development, husbandry, animal welfare, and collaboration with governmental authorities [44].

Research across Psittaciformes identifies recurring trade-related concerns involving extraction practices, supply-chain actors, transport routes, consumer demand, enforcement limitations, and intervention design [45]. Commercial captive breeding may reduce demand for wild-caught parrots when production is transparent and verifiable, but it may also facilitate laundering when wild-caught birds are misdeclared or supporting documentation is unreliable [46,47,48]. Differences between domestic and international regulations in Neotropical countries further complicate enforcement because possession, breeding, transport, and sale may be governed differently across jurisdictions [49,50]. CITES source codes and breeder declarations should therefore be evaluated alongside species biology, reproductive capacity, legal-acquisition records, physical identification, facility oversight, and chain-of-custody documentation [9,10,46,47,48,51]. Parentage testing, population assignment, and wildlife-forensic genetics can provide additional verification, although their accuracy depends on the quality and geographic coverage of reference samples [35,52,53,54].

Digital markets also create methodological and jurisdictional challenges. Studies have documented Indonesian parrot listings and broader wildlife activity on Facebook, Instagram, e-commerce sites, and other online platforms [13,14,16,18,19,20]. Automated image-recognition and content-screening tools may assist with species identification, repeated-image detection, seller-pattern analysis, and listing review, but their outputs require taxonomic validation, human assessment, ethical safeguards, and legally appropriate evidence preservation [55,56,57,58]. Direct macaw studies are concentrated in field ecology, reproduction, conservation management, and population genetics [26,27,28,29,32,33,34,35,40,41,42,43]. Peruvian market studies provide additional macaw-relevant trade context [17,39], while broader wildlife-forensic studies provide transferable methods for parentage and geographic-origin verification [52,53,54]. Broader digital-trade studies can therefore inform macaw-monitoring methods but cannot independently establish the legality, origin, or population-level effects of a particular macaw advertisement [13,14,15,16,18,19,20].

The principal gap is the fragmentation of relevant evidence. Field studies assess nesting, habitat, recruitment, and population structure [26,27,28,29,32,33,34,35]; market surveys document species availability and trade practices [17,39]; CITES analyses describe declared international transactions [9,10,11]; digital studies identify online-market signals [13,14,15,16,18,19,20,55]; and documentary or genetic methods evaluate source claims [35,46,47,48,51,52,53,54]. Because these evidence streams are generated at different spatial and institutional scales, potentially important relationships may be overlooked when they are interpreted separately. A structured method is therefore needed to connect available evidence without treating any individual indicator as definitive proof of illegality.

The purpose of this structured narrative review is to synthesize evidence concerning illegal and poorly regulated macaw trade and to propose a multiscale adaptive monitoring framework. The review distinguishes direct macaw evidence from transferable parrot evidence and broader wildlife-trade evidence so that conclusions remain proportional to the taxonomic and empirical relevance of the underlying sources. The proposed framework connects ecological and demographic monitoring, domestic and international trade records, digital-market observations, documentary and genetic source verification, welfare and post-seizure outcomes, and management responses. It is intended to support coordinated and proportionate interpretation across relevant spatial and institutional scales rather than require one researcher or organization to collect every form of evidence.

## 2. Materials and Methods

### 2.1. Review Type and Rationale

This article uses a structured narrative review to integrate heterogeneous evidence relevant to illegal and poorly regulated macaw trade. This approach is appropriate for synthesizing the literature across disciplines, study designs, source types, and levels of empirical specificity [59,60,61]. The evidence base includes species-specific ecological and demographic studies, wildlife-trade research, official databases, regulatory and policy documents, enforcement reports, animal-welfare studies, conservation-genetics research, and the digital-monitoring literature.

The review integrates ecological, regulatory, technological, forensic, welfare, and conservation-management evidence through explicit search domains, source-selection criteria, evidence coding, and thematic synthesis. Retained sources were classified as direct macaw evidence, transferable parrot evidence, or broader wildlife-trade evidence. This hierarchy allowed macaw-specific findings to be distinguished from cautious extrapolations based on other parrots and from broader contextual or methodological evidence. It was also used to identify convergence, disagreement, and uncertainty among evidence streams and to ensure that conclusions remained proportional to the taxonomic relevance and empirical strength of the supporting sources.

### 2.2. Search Strategy and Source Selection

Searches were conducted from May through July 2026 using Google Scholar, Web of Science, Scopus, ScienceDirect, SpringerLink, Wiley Online Library, Taylor & Francis Online, PubMed, the CITES Trade Database, the CITES Checklist of Species, U.S. Fish and Wildlife Service resources, UNODC reports, and selected conservation-organization publications. No strict publication-date cutoff was applied because the evidence domains examined in this review have developed at different rates.

The literature published from 2021 through 2026 was emphasized for rapidly changing topics, including online wildlife markets, automated detection, captive-bred source claims, source-verification methods, contemporary CITES interpretation, and adaptive conservation monitoring. Earlier sources were retained when they provided foundational or longitudinal evidence concerning macaw life history, nesting ecology, reproductive constraints, population dynamics, nest poaching, domestic trade, regulatory development, conservation genetics, or the interpretation of historical trade records. Publication date alone was therefore not used as an exclusion criterion.

Sources were retained when they contributed to the review objectives through direct macaw evidence, transferable evidence concerning other parrots, or broader wildlife-trade evidence relevant to monitoring and interpretation. Searches combined macaw genera and species names with terms concerning cavity use, nesting biology, reproductive success, brood reduction, chick and adult survival, breeding-population structure, population genetics, reintroduction, nest protection, conservation intervention, extraction, and domestic trade. Geographic terms included southeastern Peru, Tambopata, Costa Rica, Brazil, Bolivia, and the broader Neotropics. Earlier studies were retained when they provided longitudinal, population-level, or intervention evidence relevant to macaw ecology, demographic resilience, trade pressure, or conservation management. Table 1 summarizes the principal search domains, search combinations, purposes, and exclusion criteria.

**Figure 1:**
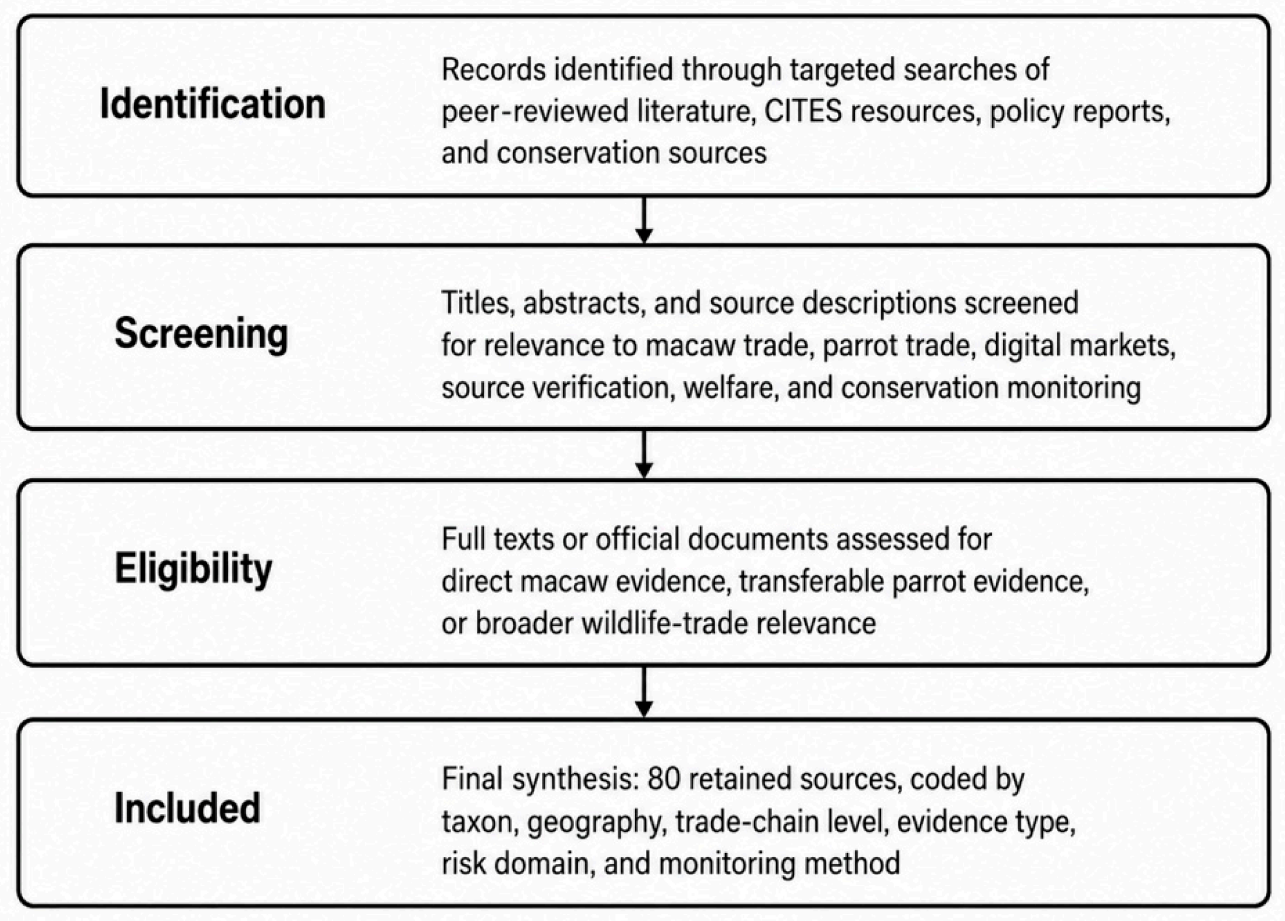
Source-accounting workflow for the structured narrative review. The figure documents how the final synthesis was organized.

After the search domains and selection criteria were established, sources were screened and organized using a staged source-accounting process. Figure 1 summarizes the workflow from source identification through final inclusion.

### 2.3. Characteristics of Retained Sources

To clarify the composition of the evidence base, retained sources were grouped by primary source category. These categories included peer-reviewed articles, official databases, agency or intergovernmental reports, policy and legal documents, and selected conservation or gray literature. Table 2 summarizes the categories represented in the final synthesis and the number of sources assigned to each category.

By primary evidentiary role, the retained sources included direct macaw evidence (n = 14), transferable parrot evidence (n = 11), and broader wildlife-trade, contextual, or methodological evidence (n = 55).

### 2.4. Evidence Coding and Evidentiary Hierarchy

Each retained source was coded by taxonomic focus, geographic region, trade-chain level, source type, risk domain, monitoring method, and relevance to forest resilience. Sources were also assigned to one of three evidentiary levels: direct macaw evidence, transferable parrot evidence, or broader wildlife-trade, contextual, or methodological evidence. These classifications guided the thematic synthesis and calibrated the strength of macaw-specific inferences. Table 3 summarizes the coding framework used in the analysis.

**Figure 2:**
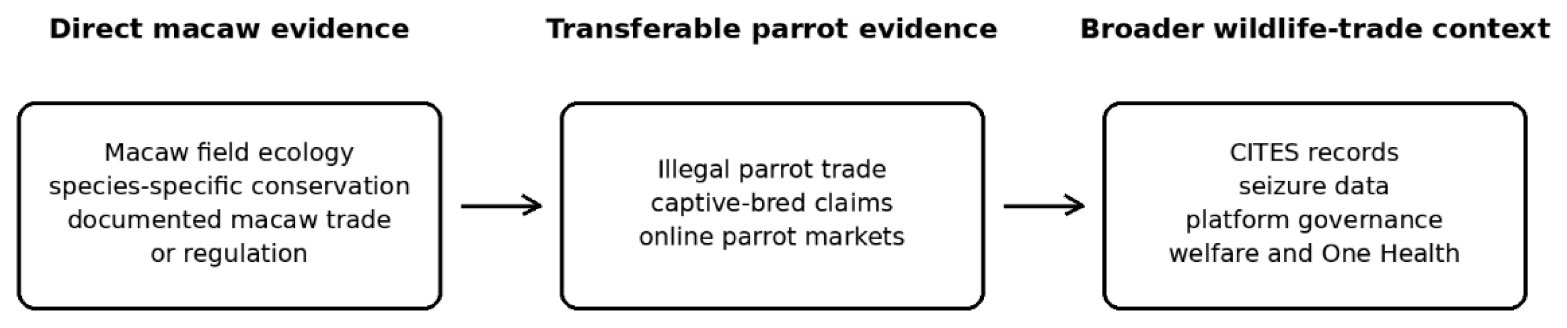
Evidentiary hierarchy used to calibrate macaw-specific conclusions. The strength and taxonomic relevance of inference decrease from direct macaw evidence to transferable parrot evidence and broader wildlife-trade, contextual, or methodological evidence.

Figure 2 illustrates the evidentiary hierarchy used to guide interpretation throughout the review. Direct macaw evidence supports the strongest macaw-specific inferences, transferable parrot evidence supports cautious extrapolation, and broader wildlife-trade, contextual, or methodological evidence informs framework development and interpretation.

### 2.5. Interpretation of Legal and Illegal Trade Evidence

A major methodological issue in wildlife-trade reviews is the relationship between legal trade records, seizure data, market observations, and illegal activity. This review treats CITES records as evidence of declared international trade, not as direct measurements of illegal trade [9,10]. Seizure data are treated as evidence of enforcement contact, not as unbiased prevalence estimates [8,12]. Online advertisements are treated as market signals requiring species identification, legal context, documentation review, and ethical handling [13,14,15,16,18]. Captive-bred claims are treated as origin claims that may be legitimate or may require verification depending on species, route, age, documentation, facility capacity, and regulatory context [46,47,48]. These interpretive rules were applied consistently in the thematic synthesis that follows.

## 3. Results: Thematic Synthesis

### 3.1. Direct Macaw Evidence: Forest Extraction, Nesting Constraints, and Recruitment Risk

Within the reviewed literature, macaw-specific studies are concentrated in ecology, demography, conservation management, genetics, and regulatory assessment [26,27,28,29,30,32,33,34,35,40,41,42,43,62], whereas direct estimates of illegal removal remain limited [17,39]. Studies of Hyacinth Macaws in Brazil identify habitat loss, limited protected-area coverage, food and nesting-resource availability, and population genetic structure as important conservation variables [26,28]. Genetic analysis has also been used to estimate the probable geographic origin of confiscated Hyacinth Macaws when suitable reference populations are available [35]. Research on Great Green Macaws shows that nest-site and landscape characteristics are associated with reproductive success in human-modified environments [27]. In southeastern Peru, long-term Scarlet Macaw studies have examined nest-site selection, artificial-nest use, brood reduction, chick mortality, foster parenting, and supplemental feeding [32,33,34].

Comparative research in southeastern Peru has also documented species-specific differences in cavity use and reproductive success among Scarlet, Red-and-green, and Blue-and-yellow Macaws [40]. Conservation interventions have been evaluated through reintroduction projects in Peru and Costa Rica [42], long-term Scarlet Macaw population monitoring in Costa Rica [41], and assessment of breeding participation and reproductive performance in Lear’s Macaws in Brazil [43]. Together, these studies identify nesting resources, breeding participation, post-release survival, and sustained conservation interventions as factors influencing population recovery, but they do not quantify current illegal-extraction prevalence across macaw species or regions.

The demographic consequences of extraction depend partly on the life stage removed [5,6,7,32,34]. Nest poaching eliminates the immediate reproductive output of breeding pairs and has been documented as a conservation pressure among Neotropical parrots [5]. Scarlet Macaw research in Peru shows that chick survival and fledging output are influenced by brood structure and may be improved through targeted management interventions under defined conditions [32,34]. Broader demographic research on long-lived birds indicates that population growth is often especially sensitive to adult survival [6,7]. Applied cautiously to macaws, this evidence suggests that removing a breeding adult may have longer-lasting effects than the loss of a single nesting attempt because it eliminates future reproductive contributions [6,7]. These effects may be amplified where habitat loss, nest-site scarcity, population fragmentation, or limited immigration already constrain recovery [6,7,26,27,28].

Across the reviewed taxa, vulnerability to removal reflects interactions among habitat condition, nesting-resource availability, reproductive output, adult survival, and population connectivity [6,7,26,27,28,32,33,34,35]. However, differences in species biology, geographic setting, study design, and conservation endpoint prevent uniform generalization across macaw populations. The strongest inference is therefore that demographic risk is species- and context-dependent, not that all macaw taxa experience equivalent extraction pressure or recovery constraints.

Direct macaw evidence therefore supports interpreting trade-related removals in relation to species-specific habitat, nesting, recruitment, adult-survival, and population-connectivity indicators rather than solely as counts of birds removed. Table 4 summarizes the principal macaw-specific evidence, its trade-relevant conservation implications, and the limits of inference for each taxon.

### 3.2. Transferable Parrot Evidence: Illegal Trade Patterns and Supply-Side Interventions

Research across Psittaciformes identifies recurring trade patterns involving wild extraction, intermediaries, transport routes, consumer demand, enforcement limitations, and intervention design [11,13,14,45,46,49,63,64]. Market surveys in Peru have also documented substantial domestic trade in parrots, including macaws and other conservation-relevant species [17,39]. Research on organizations responding to domestic wildlife-pet trade in Peru further indicates that interventions may combine outreach, advocacy, livelihood development, husbandry, animal welfare, and collaboration with governmental authorities [44]. These findings show that internal markets may operate outside international reporting systems, although the available surveys remain geographically and temporally bounded and cannot independently estimate extraction rates, identify source populations, or establish current trade prevalence across macaw taxa [9,10,17,39].

Commercial captive breeding represents a conditional supply-side intervention. Captive production may reduce demand for wild-caught parrots when breeding operations are transparent, biologically plausible, and adequately regulated, but it may also facilitate laundering when wild-caught birds are misdeclared or supporting documentation is unreliable [46,47,65]. The literature therefore does not support treating captive breeding as either inherently conservation-positive or inherently suspicious. Its potential contribution depends on species biology, breeding feasibility, founder stock, production capacity, traceability, and regulatory oversight [46,47,48,51].

These findings are relevant to macaw monitoring because macaws share biological and commercial characteristics with other traded parrots, including delayed reproduction, long life histories, market desirability, and exposure to regulated trade [5,6,7,11,26,27,28,29,33,34,36,39,45,46,49,64]. However, transferability depends on similarities in species biology, legal context, market structure, and enforcement capacity [11,45,46,47,48,49]. Parrot studies can therefore identify plausible trade mechanisms and monitoring questions, but they cannot establish equivalent trade intensity, laundering practices, or conservation effects for every macaw species or jurisdiction.

### 3.3. CITES, Source Codes, and the Legal–Illegal Interface

The CITES Trade Database provides the principal standardized record of declared international trade in CITES-listed species, including taxa, trade terms, quantities, source codes, purposes, exporters, and importers [9,10,66]. These records can reveal longitudinal changes in declared trade volumes, shifts in source-code use, and recurring exporter–importer relationships [9,10,11,67]. However, they document reported international transactions rather than the full extent of wildlife commerce and do not capture all domestic trade, undetected extraction, unreported trafficking, or variation in enforcement and reporting effort [8,9,10,11,12]. The absence of a CITES record therefore does not establish that trade is absent, while increasing declared trade does not independently demonstrate illegal activity [8,9,10,11,12].

For macaws, source codes should be interpreted in relation to species biology and the plausibility of the declared origin. Many macaw species are long-lived, mature slowly, reproduce at comparatively low rates, and depend on suitable nesting resources [5,6,7,26,27,28,29,30,32,33,34]. Declared captive-bred, born-in-captivity, wild-sourced, ranched, unknown, or confiscated origins may therefore carry different conservation implications, but no source code should be evaluated in isolation [9,10,51]. Relevant comparisons include species and age, trade term, declared purpose, founder stock, reproductive timelines, breeder capacity, legal-acquisition records, facility oversight, physical identification, and chain-of-custody documentation [46,47,48,51].

Genetic methods can provide an additional verification layer when documentary evidence is incomplete or inconsistent [35,47,52,53,54]. Parentage testing may assess whether a bird declared as captive-bred is genetically compatible with the claimed breeding adults, while population-assignment methods may help estimate the probable geographic origin of confiscated individuals [35,52,53,54]. These methods cannot independently establish legality and depend on validated markers, representative reference samples, laboratory quality, and secure chains of custody [35,52,53,54]. Their evidentiary value is strongest when genetic findings are interpreted alongside breeder records, permit review, physical markings, facility inspections, and enforcement information [35,47,48,51,52,53,54].

CITES records and source codes should therefore be treated as monitoring and verification signals rather than as direct measurements of illegal trade. Unusual declared volumes, abrupt source-code changes, biologically implausible production levels, or inconsistencies among documentary, biological, and genetic evidence may justify additional review, but each pattern may also have lawful or administrative explanations [8,9,10,11,12,35,46,47,48,51,52,53,54]. Table 5 compares the evidentiary strengths and limitations of the principal monitoring approaches and shows how the proposed framework connects their outputs.

### 3.4. Digital-Market Risk and Online Wildlife Trade

Digital wildlife trade occurs across public advertisements, social-media groups, e-commerce platforms, temporary posts, coded terminology, and private communication channels [13,14,15,16,18,19,20]. Research concerning Indonesian parrots and mixed wildlife taxa indicates that online platforms can broaden market reach while complicating species identification, seller attribution, evidence preservation, legal interpretation, and ethical data collection [13,14,15,16,18]. Automated image-recognition and content-screening methods may assist with species identification, repeated-image detection, seller-pattern analysis, and recurring-terminology detection, but their outputs require expert validation and human review [55,56,57,58].

Because macaw-specific digital-trade research remains limited, methods developed through studies of other parrots and mixed wildlife taxa should be treated as transferable monitoring approaches rather than as direct evidence of macaw-trade prevalence [13,14,15,16,18,19,20]. Indicators such as repeated seller activity, image reuse, rare-species claims, inconsistent documentation, and unclear origin information may help prioritize listings or seller networks for further assessment [13,14,15,16,18]. Interpretation should incorporate expert species validation, legal-context review, ethical collection of publicly accessible information, secure evidence preservation, human assessment of automated outputs, and appropriately authorized referral procedures [15,16,18,55,56,57,58].

### 3.5. Animal Welfare, One Health, and Post-Seizure Conservation Outcomes

Live wildlife trafficking may expose animals to capture stress, dehydration, injury, crowding, disease, social deprivation, and mortality during transport or confinement [72,73]. Welfare concerns may persist after confiscation because animals can require veterinary treatment, disease screening, behavioral rehabilitation, sanctuary placement, or evaluation for release [71,73,74]. Confiscation therefore represents an enforcement event rather than a complete conservation outcome; it does not establish that an animal survived, recovered, reproduced, or was successfully returned to a wild population [71,73,74].

Informal movement of live wildlife may also alter human–animal interfaces and create pathogen-transmission and biosecurity risks [72,75]. Trade-related escape or release of captive parrots may also create downstream ecological effects outside their native ranges [76]. The reviewed evidence does not identify macaws as uniquely important disease vectors. Instead, it links uncertain animal origin, poor transport conditions, close confinement, and limited post-seizure capacity with interconnected welfare and health concerns [72,73]. Evaluation should therefore extend beyond confiscation totals to include survival, disease status, rehabilitation outcomes, release suitability, sanctuary capacity, and longer-term contributions to conservation recovery [71,73,74].

### 3.6. Synthesis: Illegal Macaw Trade as a Layered Risk System

The reviewed literature indicates that illegal and poorly regulated macaw trade operates across connected ecological, commercial, regulatory, digital, verification, and post-seizure contexts. Field studies document habitat conditions, nesting-resource availability, reproductive performance, chick survival, and population structure [26,27,28,32,33,34,35]. Physical-market studies identify domestic availability of parrots, including macaws, at particular locations and times [17,39]. CITES records and enforcement datasets describe declared international transactions and detected trafficking activity, but they do not capture all domestic commerce, undetected extraction, or unreported trade [8,9,10,11,12]. Digital-market studies identify online listings, seller activity, image reuse, terminology, and movement across platforms, although species identity, origin, documentation, and legality may remain uncertain [13,14,15,16,18,19,20].

These evidence streams represent different points within a layered risk system. Extraction occurs within ecological conditions that influence whether populations can compensate for the loss of eggs, chicks, or breeding adults [5,6,7,26,27,28,32,33,34]. Birds may then move through intermediaries, physical markets, breeding facilities, declared international trade, or online networks [8,9,10,11,12,13,14,15,16,17,18,19,20,39,45,46,47,49]. Captive-bred and geographic-origin claims introduce an additional verification layer involving legal-acquisition records, reproductive plausibility, facility capacity, physical identification, and genetic evidence [35,46,47,48,51,52,53,54]. When animals are confiscated, welfare, rehabilitation, release suitability, sanctuary capacity, and longer-term conservation outcomes become part of the same system [71,72,73,74].

No individual evidence stream can independently characterize the entire trade pathway or establish the legality of a particular animal, transaction, seller, or facility. Ecological decline may have causes unrelated to trade, declared transactions may be lawful, seizures reflect enforcement contact, online listings may be misidentified, and genetic findings require documentary and investigative context [8,9,10,12,13,14,15,16,18,35,52,53,54]. The principal synthesis is therefore that macaw-trade risk should be evaluated through relationships among evidence streams rather than through isolated indicators. This finding provides the basis for the multiscale adaptive monitoring framework presented in Section 4.1.

## 4. Discussion

### 4.1. Novelty and Added Value of the Adaptive Monitoring Framework

Adaptive management and adaptive monitoring are established conservation approaches in which monitoring results are used to evaluate assumptions, revise indicators, and adjust management actions over time [77,78]. The novelty of the proposed framework does not lie in creating new field, trade-record, digital, documentary, or genetic methods. Rather, it applies adaptive-monitoring principles to macaw trade as a connected forest-to-market problem spanning ecological, regulatory, commercial, forensic, welfare, and institutional scales [2,3,4,45,77,78].

**Figure 3:**
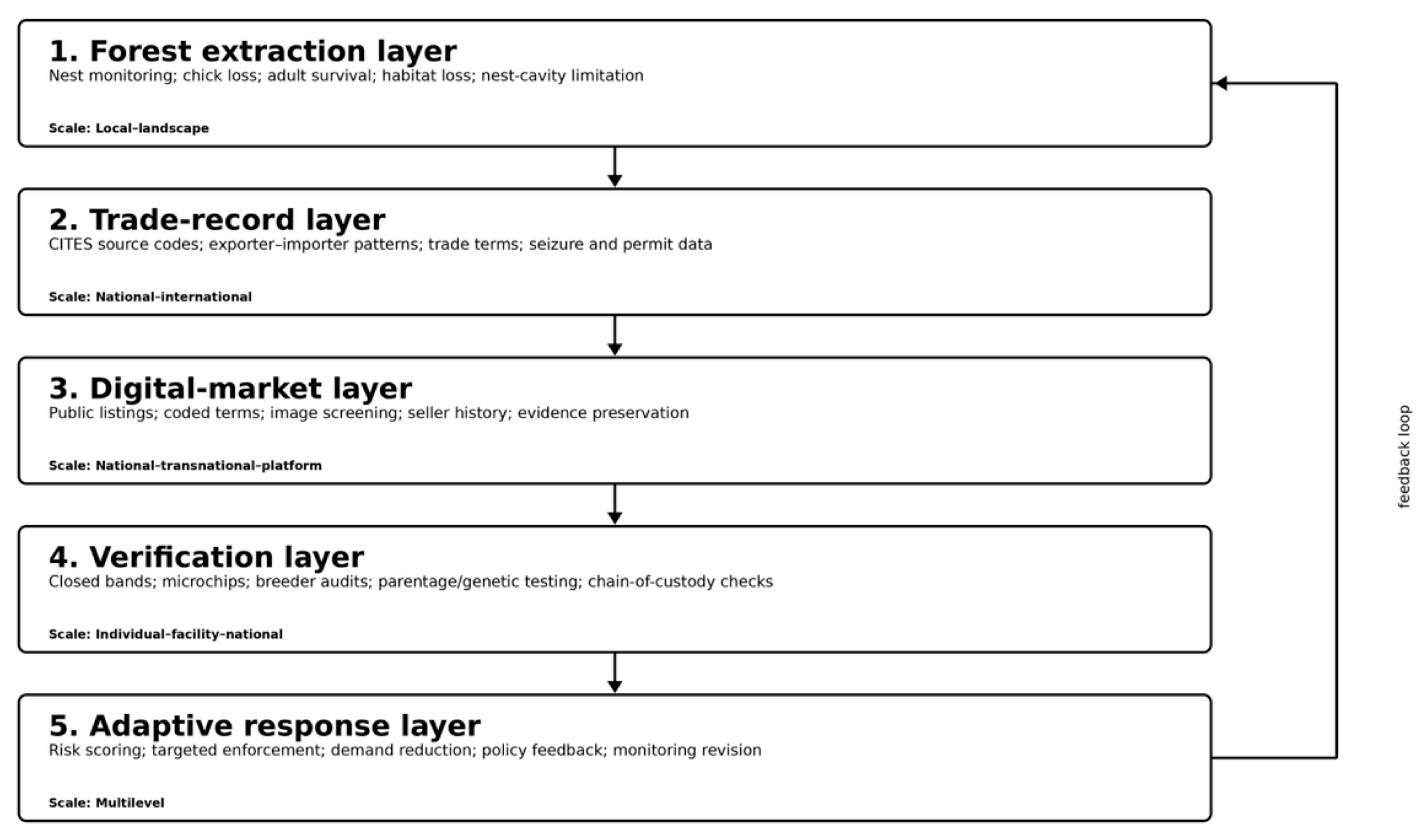
Multiscale adaptive monitoring framework for macaw trade risk. The framework integrates ecological, trade, digital-market, and source-verification evidence to guide authorized adaptive responses. Monitoring outcomes inform revisions to indicators, verification priorities, and management actions.

Existing approaches generally examine field ecology, declared trade, enforcement encounters, digital markets, or source verification separately. The framework’s contribution is to organize their outputs around a defined conservation question and an iterative process of monitoring, verification, response, and evaluation [8,9,10,11,12,13,14,15,16,17,18,19,20,26,27,28,32,33,34,35,39,45,46,47,48,49,51,52,53,54,77,78]. Figure 3 presents this process as a feedback cycle connecting monitoring, verification, and proportionate management responses.

By comparing outputs across evidence streams, the framework can identify convergence, contradiction, and evidentiary gaps without treating any individual indicator as conclusive [8,9,10,11,12,13,14,15,16,18,19,20,35,45,46,47,48,52,53,54]. Agreement among independent indicators may strengthen the justification for additional verification, whereas disagreement may reflect lawful trade, domestic commerce, reporting gaps, variable enforcement effort, species misidentification, incomplete documentation, or limitations in available genetic reference data [8,9,10,11,12,35,46,47,48,49,52,53,54].

For example, low seizure frequency cannot independently establish limited trade because seizure patterns are influenced by enforcement effort and detection capacity [8,12]. However, low seizure frequency combined with repeated online availability, increasing captive-bred declarations, and limited documented breeding capacity may justify breeder-record review, facility inspection, parentage testing, or focused trade-route analysis [13,14,15,16,35,46,47,48,52,53,54]. Conversely, declared trade may present less immediate conservation concern when source codes, founder stock, reproductive timelines, facility capacity, legal-acquisition records, and genetic findings consistently support captive production [9,10,11,35,46,47,48,51,52,53,54].

### 4.2. Implementation and Evaluation

The framework is intended for conservation programs, wildlife authorities, CITES authorities, researchers, enforcement agencies, nongovernmental organizations, and veterinary or genetic laboratories operating within their respective mandates [9,10,15,35,44,48,51,52,53,54,79]. Implementation begins by defining the focal species or population, conservation question, geographic and temporal scope, jurisdiction, participating organizations, available evidence streams, and thresholds for further action [77,78]. Responsibilities may then be distributed among institutions rather than assigned to a single organization.

Evidence selection should reflect the question being examined. Field programs may monitor habitat condition, nest disturbance, reproductive performance, and recruitment [26,27,28,32,33,34]. Regulatory and enforcement authorities may assess permits, declared trade, source codes, and seizures [8,9,10,11,12]. Digital monitoring requires ethical collection, secure preservation, reliable species identification, and authorized referral procedures [13,14,15,16,18,19,20], while source verification may incorporate breeder records, physical identification, veterinary documentation, parentage testing, and geographic-origin assignment [35,46,47,48,51,52,53,54].

Responses should remain proportional to the strength and convergence of the evidence. Weak or isolated indicators may support continued monitoring, whereas repeated inconsistencies among ecological conditions, trade records, online activity, breeder capacity, documentation, and genetic findings may justify record review, facility inspection, targeted testing, or authorized investigation [8,12,15,35,45,46,47,48,51,52,53,54].

Adaptive implementation also requires evaluating whether interventions achieve their intended outcomes [77,78]. Nest protection may be assessed through occupancy, reproductive success, and chick survival [27,32,33,34]; breeder verification through improved documentation and traceability [46,47,48,51]; and digital interventions through changes in observable listings or movement across platforms [13,14,15,16,18,19,20]. These findings should inform subsequent revisions to indicators, decision thresholds, monitoring priorities, and management actions [77,78].

### 4.3. Illustrative Applications of the Framework

The following hypothetical scenarios illustrate how the framework could guide verification and adaptive management. They are not empirical tests and do not imply that any described activity is illegal [2,3,4,45,77,78].

**Scenario 1: Declining Nest Productivity and Regional Market Signals**

Monitoring may identify declining nest occupancy, chick survival, or fledgling production within a defined macaw population [27,32,33,34]. Because habitat disturbance, nest-site limitation, predation, brood reduction, and natural variation may produce similar patterns, ecological decline alone cannot establish extraction [26,27,28,32,33,34]. However, concurrent public advertisements for juvenile birds in the surrounding region may justify species and age validation, review of seller histories and origin claims, examination of nest disturbance, and consultation with wildlife authorities [13,14,15,16,18]. Nest protection may also be prioritized where ecological vulnerability and market signals overlap [5,27,32,33,34].

**Scenario 2: Increasing Captive-Bred Exports**

CITES records may show increasing exports under captive-bred source codes [9,10,11]. This pattern may reflect legitimate production, but further verification may be appropriate when declared quantities, age classes, or export frequency appear inconsistent with founder stock, reproductive timelines, facility inventories, or breeding capacity [46,47,48,51]. Authorized authorities could compare permits with breeding records, hatch dates, physical markings, veterinary documents, and inspection findings. Parentage testing may evaluate claimed breeding relationships, while geographic-assignment methods may assist when wild origin is suspected and suitable reference data are available [35,52,53,54].

**Scenario 3: Low Seizure Frequency in a Vulnerable Population**

A macaw population may occupy fragmented habitat or experience limited nesting resources while appearing infrequently in seizure records [12,26,27,28]. Low seizure frequency may indicate limited trafficking, but it may also reflect weak detection, domestic commerce, unmonitored routes, or trade outside international reporting systems [8,9,10,11,12,17,39]. Field indicators can therefore be compared with domestic-market surveys, public listings, permits, community observations, and enforcement capacity [12,13,14,15,16,17,18,26,27,28,32,33,34,39]. Weak evidence may support continued monitoring, whereas convergence may justify nest protection, market observation, community engagement, documentation review, or targeted patrols [2,3,4,45,77,78].

These three scenarios demonstrate how incomplete evidence can be compared, assigned to appropriate institutions, and matched with proportionate verification or management responses [2,3,4,45,77,78]; the following section defines the limits of the inferences that can be drawn from the underlying evidence.

### 4.4. Proportional Interpretation of Evidence

Direct macaw studies support species-specific ecological and conservation inferences [26,27,28,29,32,33,34,35,40,41,42,43]; parrot studies support cautious extrapolation [11,13,14,17,39,45,46,47,49,63,64]; and broader wildlife-trade research mainly informs monitoring and verification methods [8,12,13,14,15,16,18,19,20,44,52,53,54,55,58,71,72,73,74]. These evidence levels are not equivalent measures of macaw-trade intensity or illegality. Converging indicators may justify further verification, but lawful trade, habitat disturbance, domestic commerce, misidentification, reporting error, and incomplete documentation remain plausible alternatives [8,9,10,11,12,15,35,45,46,47,48,49,52,53,54]. Accordingly, the evidence supports species-specific monitoring of a plausible conservation risk, not a global prevalence estimate or presumption of unlawful activity.

### 4.5. Research Gaps and Future Directions

These interpretive limits also define the principal priorities for future empirical research. The synthesis identifies persistent gaps in species-specific estimates of trade prevalence, verification of captive-bred claims, macaw-specific digital monitoring, demographic effects of extraction, and post-seizure outcomes [5,6,7,11,12,13,14,17,26,27,28,32,33,34,35,39,45,46,47,49,52,53,54,71,73,74,80]. It also highlights the need to empirically evaluate the proposed framework and refine its indicators, thresholds, and feedback processes [77,78]. Table 6 summarizes how each gap limits current inference and identifies focused priorities for future research.

### 4.6. Limitations

These priorities should be interpreted in light of the limitations of the present structured narrative review and proposed framework, which may not have captured all relevant evidence. Regional reports, non-indexed publications, confidential enforcement records, and unpublished conservation data may have been missed [59,60,61]. Although the explicit search, selection, and coding procedures improve transparency, they do not provide the comprehensiveness of a systematic review or the quantitative comparability required for meta-analysis [59,60,61].

The evidence base is uneven across macaw species, regions, and trade pathways. Direct macaw studies are concentrated in habitat, nesting ecology, reproduction, conservation interventions, and population genetics, whereas species-specific evidence concerning illegal extraction, domestic markets, online trade, and laundering remains limited [17,26,27,28,29,32,33,34,35,39]. Findings from well-studied taxa or locations therefore should not be generalized across all macaw populations without additional species- and region-specific evidence.

Several evidence streams are indirect or incomplete. CITES records document declared international transactions, seizures reflect enforcement contact, and online advertisements may involve lawful activity, inaccurate identifications, or unverifiable origin claims [8,9,10,11,12,13,14,15,16,18,19,20]. Ecological changes may also result from habitat degradation, resource limitation, predation, disease, climate, or natural reproductive variation rather than extraction [26,27,28,32,33,34]. The framework may consequently produce false-positive or false-negative interpretations when indicators are poorly defined, dependent on one another, or interpreted outside their ecological and legal contexts [2,3,4,15,45,48]. Applications should therefore predefine indicators, document data quality, consider alternative explanations, and establish transparent thresholds for additional verification [77,78].

Implementation may be constrained by institutional authority, data access, funding, technical capacity, and restrictions on sharing confidential or jurisdictionally sensitive information [2,15,48,51,52,53,54]. Genetic testing, breeder audits, digital investigations, secure data management, and long-term ecological monitoring may also be impractical in resource-limited settings [15,35,47,52,53,54]. Online monitoring introduces additional ethical and legal concerns involving personal information, private groups, deleted content, suspected criminal activity, and cross-jurisdictional evidence [13,14,15,16,18,19,20]. Digital applications should therefore use approved protocols, secure evidence handling, expert species validation, and appropriately authorized referral procedures [15,16,18,55,56,57,58].

Finally, the proposed framework and illustrative scenarios remain conceptual rather than empirically validated. They organize available evidence but do not establish improved detection, enforcement efficiency, or conservation outcomes [77,78]. Because several recommendations rely on transferable parrot or broader wildlife-trade evidence, they should not be interpreted as demonstrating equivalent conditions across macaw species or jurisdictions [2,3,4,11,12,13,14,45,46,47,49]. The framework is therefore presented as a testable and adaptable decision-support structure rather than a validated universal protocol.

## 5. Conclusions

Illegal and poorly regulated macaw trade intersects forest ecology, population dynamics, animal welfare, legal commerce, digital markets, source verification, and conservation governance [2,3,4,5,6,7,26,27,28,35,45,47,48,52,53,54,71,72,73,74]. The reviewed evidence does not support a single global estimate of illegal-trade prevalence or equivalent risk across macaw species, populations, regions, and trade pathways [11,17,26,27,28,29,32,33,34,35,39,45,46,49]. It does indicate that extraction may be particularly consequential where habitat loss, limited nesting resources, low reproductive output, population fragmentation, and restricted recovery capacity already constrain populations [5,6,7,26,27,28,32,33,34,35].

The principal contribution of this review is a multiscale adaptive monitoring framework that connects ecological indicators, trade and enforcement records, digital-market observations, source verification, and management responses. The framework coordinates existing evidence streams around a defined species, population, facility, route, jurisdiction, or market rather than requiring one institution to implement every component [2,3,4,15,35,45,48,51,52,53,54,77,78]. Convergence among independent indicators may justify proportionate verification, but no ecological, commercial, documentary, genetic, or enforcement signal independently establishes illegality [8,9,10,11,12,13,14,15,16,18,19,20,35,45,46,47,48,49,52,53,54]. Pending species- and jurisdiction-specific validation, illegal macaw trade should be treated as a plausible and context-dependent conservation stressor requiring integrated, cautious, and adaptive monitoring [77,78].

## Figures and Tables

**Table 1:** Search strategy and selection criteria for the structured narrative review.

Search Domain	Core Search Combinations	Purpose	Main Exclusion Criteria
Macaw and parrot trade	(macaw OR parrot OR Psittaciformes OR Ara OR Anodorhynchus OR Cyanopsitta OR Primolius) AND (“illegal trade” OR trafficking OR poaching OR extraction OR “domestic trade” OR “internal trade”), combined where relevant with (Peru OR “Peruvian Amazon” OR Tambopata OR “southeastern Peru” OR Neotropics)	Identify direct macaw evidence and broader Psittaciformes trade patterns and regional market studies.	Pet-care, husbandry, or aviculture sources without conservation, trade, regulatory, or enforcement relevance.
CITES and legal-trade interface	(parrot OR macaw) AND (CITES OR “source code” OR “captive-bred” OR laundering OR “legal acquisition” OR traceability OR “breeder capacity”)	Identify evidence on declared trade, source codes, verification, captive-bred claims, documentation, and legal–illegal ambiguity.	General CITES commentary without relevance to birds, live-animal trade, source claims, or traceability.
Digital wildlife trade	(“wildlife trade” OR “exotic pet trade” OR parrot OR macaw) AND (online OR “social media” OR Facebook OR Instagram OR “e-commerce” OR marketplace OR “online marketplace” OR “private messaging”)	Identify evidence on platform-mediated wildlife trade, online-market indicators, and digital monitoring methods.	Non-wildlife cybercrime or e-commerce studies without conservation or wildlife-trade relevance.
Macaw ecology and population dynamics	(macaw OR parrot OR *Ara* OR *Anodorhynchus*) AND (“nest poaching” OR recruitment OR “nest-site limitation” OR “nest-site selection” OR “reproductive success” OR “chick survival” OR “brood reduction” OR “adult survival” OR “habitat connectivity” OR “population viability” OR “cavity use” OR “breeding population” OR “breeding performance” OR reintroduction OR “nest protection” OR “conservation intervention” OR “population genetics”) AND (“southeastern Peru” OR Tambopata OR “Costa Rica” OR Brazil OR Bolivia OR Neotropics)	Connect extraction to reproductive ecology, demographic sensitivity, habitat constraints, population genetics, population resilience, and conservation interventions across major Neotropical macaw research regions.	Captive-only behavior or husbandry studies without relevance to wild populations, reproduction, or conservation planning.
Monitoring and source verification	(“wildlife trafficking” OR “wildlife trade” OR macaw OR parrot) AND (“machine learning” OR “image recognition” OR “source verification” OR “genetic forensics” OR “DNA forensics” OR “parentage testing” OR “population assignment” OR “breeder audit” OR “chain of custody”)	Identify tools that may strengthen detection, species identification, origin verification, captive-bred validation, traceability, and management feedback.	Technical methods papers without wildlife-trade, forensic, regulatory, or conservation application.
Welfare and post-seizure outcomes	(“wildlife trade” OR trafficking OR confiscation OR seizure) AND (“animal welfare” OR rehabilitation OR release OR sanctuary OR mortality OR disease OR “post-seizure”)	Identify welfare consequences and evaluate whether confiscation, rehabilitation, or release contributes to conservation recovery.	General captive-animal welfare studies without relevance to trafficking, confiscation, rehabilitation, or conservation outcomes.
Adaptive monitoring and management	(“adaptive monitoring” OR “adaptive management”) AND (conservation OR wildlife OR biodiversity OR “management response”)	Identify established principles for revising indicators, monitoring priorities, decision thresholds, and management responses as new evidence becomes available.	General organizational, business, or project-management literature without a conservation, wildlife, or environmental-monitoring application.

**Table 2:** Characteristics of sources retained for synthesis.

Source Category	Description	n
Peer-reviewed articles	Empirical and review literature concerning macaw and parrot ecology, population dynamics, wildlife trade, conservation genetics, animal welfare, digital monitoring, source verification, and adaptive monitoring	64
Official databases	CITES and related trade or species-status resources	2
Agency/intergovernmental reports	Wildlife-crime, biodiversity, enforcement, trade-monitoring, and adaptive-management reports and technical guidance	5
Policy/legal documents	Legal-acquisition guidance, species listings, regulatory decisions, and international policy frameworks	5
Conservation reports/gray literature	Conservation and platform-monitoring reports and selected preprints concerning digital wildlife-trade detection	4
Total		80

Note: Counts reflect the primary function of each source within the synthesis. Although individual sources sometimes informed multiple themes, each source was assigned to one primary source category to prevent duplicate counting.

**Table 3:** Evidence-coding framework used to classify retained sources and support thematic synthesis.

Coding Variable	Coding Options	Use in Synthesis
Taxonomic focus	Macaw; non-macaw parrot; mixed wildlife taxa; non-taxonomic or methodological	Identifies the taxonomic scope of each source and prevents findings from broader taxa from being treated as macaw-specific evidence.
Evidentiary level	Direct macaw evidence; transferable parrot evidence; broader wildlife-trade, contextual, or methodological evidence	Calibrates the strength of macaw-specific conclusions according to the relevance of the supporting evidence.
Geographic region	Peru/Peruvian Amazon; other Neotropics; Indonesia/Asia; global or multiregional; other national case study; unspecified or not applicable	Distinguishes evidence from focal macaw range states and Peruvian study areas from findings concerning other regions or global trade systems.
Trade-chain level	Supply or extraction; transport or intermediary; domestic or physical market; declared international or legal trade; online or digital market; demand; breeding facility or source verification; post-seizure	Shows where evidence is concentrated across the trade chain and identifies underrepresented monitoring stages.
Source type	Peer-reviewed article; official database; agency or intergovernmental report; policy or legal document; conservation report; preprint or other gray literature	Clarifies whether evidence is empirical, administrative, legal, methodological, or conceptual.
Risk domain	Forest ecology and demography; legal–illegal interface; digital market; source verification or laundering; welfare or One Health; governance or enforcement; post-seizure conservation	Links each source to the principal conservation and trade risks examined in the framework.
Monitoring method	Field ecological or demographic monitoring; trade-record or CITES analysis; physical-market survey; seizure or enforcement-data analysis; online observation; automated detection; documentary or facility verification; genetic or forensic verification; welfare or post-seizure monitoring; adaptive monitoring or evaluation	Identifies available monitoring tools, methodological gaps, and approaches requiring additional validation.
Relevance to forest resilience	Direct ecological relevance; indirect trade or governance relevance; contextual or methodological relevance	Indicates whether a source directly addresses population or ecological resilience, examines pressures affecting resilience, or contributes methods for interpreting those pressures.

**Table 4:** Macaw evidence and trade-relevant conservation concerns used to guide species-specific interpretation.

Macaw Taxon or Evidence Group	Evidence in the Reviewed Literature	Trade-Relevant Conservation Concern	Interpretation Caution
Hyacinth Macaw (*Anodorhynchus hyacinthinus*)	Studies document habitat loss, limited protected-area coverage, nesting and food-resource constraints, population genetic structure, and conservation priorities [26,28,35].	Extraction may intensify existing habitat and connectivity constraints [26,28]. Genetic assignment may help identify the probable geographic origin of confiscated birds when suitable reference populations are available [35,52,53,54].	The reviewed studies support ecological and genetic vulnerability but do not estimate current illegal-extraction prevalence [26,28,35].
Great Green Macaw (*Ara ambiguus*)	Nest-site and landscape characteristics are associated with reproductive success in human-modified habitats [27].	Removal may have greater demographic effects where suitable cavities and reproductive output are limited [5,6,7,27].	The available study does not measure current illegal-trade pressure [27]. Assessing such pressure would also require market, enforcement, and source-verification evidence [12,17,35,39,52,53,54].
Scarlet Macaw (*Ara macao*)	Peruvian research documents nesting ecology, brood reduction, chick mortality, and interventions using artificial nests, foster parents, and supplemental feeding [32,33,34,40,41,42]. Behavioral and regulatory studies provide additional context [29,30,62].	Nest removal can eliminate immediate reproductive output [5], while targeted interventions may improve chick survival and fledging success under defined conditions [32,33,34,40,41,42].	These studies do not quantify illegal extraction across the species’ range [29,30,32,33,34,40,41,42,62], and legal status and regulatory conditions vary among jurisdictions [30,49,62].
Lear’s Macaw (*Anodorhynchus leari*)	Population monitoring distinguishes breeding from non-breeding individuals and documents variation in breeding performance [43].	A limited breeding fraction or low reproductive output may restrict population recovery following the removal of chicks or breeding adults [6,7,43].	The study informs demographic monitoring but does not estimate illegal-extraction prevalence or attribute observed population patterns directly to trade [43].
Cross-species macaw synthesis	Direct studies identify habitat condition, nesting-resource availability, reproductive constraints, and population structure as important conservation variables [26,27,28,29,32,33,34,35,40].	Removals should be interpreted in relation to the life stage removed, the demographic importance of affected individuals, and source-population recovery capacity [5,6,7,26,27,28,32,33,34,40].	Findings cannot be generalized uniformly because ecology, population status, legal protection, and trade exposure differ among taxa and regions [26,27,28,29,30,32,33,34,35,40,49,62].

**Table 5:** Strengths, limitations, and integration of monitoring approaches relevant to illegal macaw trade.

Existing Monitoring Approach	Strengths	Limitations	How the Proposed Framework Integrates the Approach
CITES declared trade analysis	Provides standardized international records of reported taxa, quantities, source codes, purposes, and exporter–importer relationships [9,10,11].	Does not directly measure illegal or domestic trade and depends on the completeness and accuracy of national reporting [8,9,10,11].	Compares declared volumes, source-code patterns, purposes, and routes with field, market, documentary, and genetic evidence. Discrepancies are treated as grounds for additional verification rather than as proof of illegality [9,10,35,46,47,48,51,52,53,54].
Seizure and enforcement datasets	Document enforcement contact, confiscated species, detected routes, and intended uses [8,12].	Reflect enforcement effort and detection capacity as well as trafficking activity; few seizures do not necessarily indicate low trade pressure [8,12].	Interprets seizures in relation to enforcement effort and compares them with ecological observations, declared trade, physical-market surveys, and digital-market indicators [8,9,10,12,13,14,15,16,17,18,19,20,39].
Field nest and habitat monitoring	Measures nest occupancy, reproductive output, chick survival, habitat condition, and other population-relevant ecological indicators [26,27,28,32,33,34].	Field monitoring documents local ecological change, but cannot independently identify sellers, routes, source claims, legal status, or online activity [26,27,28,32,33,34].	Connects local ecological and demographic indicators with trade records, market observations, source documentation, and authorized enforcement information [8,9,10,12,17,39].
Online market monitoring	Can identify dispersed listings, repeated seller activity, species availability, image reuse, movement across platforms, and market drivers [13,14,15,16,18,19,20,24,68,69,70].	Species identity, age, geographic origin, legality, seller intent, and documentation may remain uncertain; activity may move into private or temporary channels [13,14,15,16,18].	Requires ethical data collection, expert species validation, secure evidence preservation, legal-context review, human assessment of automated outputs, and comparison with independent evidence before referral or intervention [15,16,18,55,56,57,58].
Captive-bred documentation	Breeder records, physical markings, veterinary documents, and production records may support lawful-origin claims when complete and verifiable [46,47,48,51].	Documentation may be incomplete, falsified, or inconsistent with founder stock, reproductive timelines, facility inventories, or production capacity [46,47,48].	Compares documentation with species biology, breeder capacity, facility inspections, closed bands or microchips, chain-of-custody records, and targeted genetic testing [35,46,47,48,51,52,53,54].
Conservation genetics and wildlife-forensic verification	Parentage testing may evaluate captive-bred claims, while population-assignment methods may help identify the probable geographic origin of confiscated individuals [35,52,53,54].	Reliability depends on validated markers, representative reference samples, laboratory quality, secure chain-of-custody procedures, and sufficient geographic differentiation. Genetic evidence alone does not establish legality [35,52,53,54].	Uses genetic findings as an independent verification layer when documentary, biological, or geographic claims conflict and interprets them alongside breeder audits, permit review, seizure investigations, and post-seizure decisions [35,47,48,51,52,53,54,71].
Welfare and post-seizure monitoring	Documents mortality, disease, injury, rehabilitation, release suitability, sanctuary placement, and whether confiscated animals can contribute to conservation recovery [71,72,73,74].	Confiscation totals do not show whether animals survive, recover, are appropriately released, or regain ecological or reproductive function [71,73,74].	Extends evaluation beyond enforcement activity by incorporating veterinary outcomes, rehabilitation, genetic assignment, release decisions, sanctuary placement, and longer-term conservation outcomes [35,52,53,54,71,72,73,74].

Note: The limitations describe the evidentiary boundaries of each monitoring approach rather than deficiencies in the methods themselves. The proposed framework does not replace these approaches; it connects their outputs across ecological, regulatory, market, forensic, welfare, and management contexts.

**Table 6:** Research gaps and priorities for future research.

Research Gap	Why It Matters	Recommended Next Step
Macaw-specific illegal trade prevalence	Current evidence does not support comprehensive estimates across species, regions, or trade pathways, including within Peru [17,26,27,28,29,32,33,34,35,39].	Conduct species-specific studies integrating field observations, domestic-market surveys, CITES records, seizure data, and population-status indicators [8,9,10,11,12,17,26,27,28,29,32,33,34,39].
Captive-bred verification	Captive-bred claims may represent lawful production but can also facilitate laundering when documentation or oversight is inadequate [46,47,48].	Compare founder stock, reproductive timelines, facility capacity, inventories, physical markings, and chain-of-custody records with targeted parentage or geographic-origin testing [35,46,47,48,51,52,53,54].
Online market evidence	Digital listings can reveal market signals but may not establish legality, origin, species identity, or seller intent [13,14,15,16,18,19,20].	Develop ethical online-monitoring protocols using public data, non-engagement, secure storage, expert species verification, human validation, and authorized referral procedures [15,16,18,55,56,57,58].
Population-level impacts	Removal of chicks or adults may affect recruitment and population growth, but species- and population-specific demographic consequences remain insufficiently quantified [5,6,7,26,27,28,32,33,34].	Link nest monitoring, survival estimates, habitat conditions, demographic models, and trade-risk indicators for defined populations [5,6,7,26,27,28,32,33,34].
Post-seizure conservation outcomes	Confiscation does not establish survival, recovery, release suitability, or restoration of ecological and reproductive function [71,72,73,74].	Track mortality, disease status, rehabilitation, genetic or geographic assignment, release decisions, sanctuary placement, and post-release outcomes where feasible [35,52,53,54,71,72,73,74].
Adaptive framework validation	The proposed framework remains conceptual and requires empirical evaluation as a monitoring and decision-support process [77,78].	Apply it to defined species, populations, facilities, or trade pathways using predefined indicators and evaluate evidence convergence, decision outcomes, intervention effects, and subsequent refinement [77,78].

## Data Availability

No new primary data were generated or analyzed in this review. All sources discussed are available through the cited publications, public databases, or official reports.

## Abbreviations

CITES	Convention on International Trade in Endangered Species of Wild Fauna and Flora
IUCN	International Union for Conservation of Nature
UNODC	United Nations Office on Drugs and Crime
UNEP-WCMC	United Nations Environment Programme World Conservation Monitoring Centre
